# Improving transitions between nursing homes and emergency departments: a qualitative study

**DOI:** 10.1186/s12912-025-04121-6

**Published:** 2025-12-23

**Authors:** Elin Høyvik, Malcolm Bray Doupe, Frode Fadnes Jacobsen

**Affiliations:** 1 Centre for Care Research, Western Norway, University of Applied Sciences, Bergen, Norway; 2 University of Manitoba, Winnipeg, Canada; 3 VID Specialized University, Bergen, Norway

**Keywords:** Transition, Nursing home, Emergency department, Out-of-hours primary care, Ambulance, Nursing home resident

## Abstract

**Background:**

Nursing home residents with acute illnesses have complex healthcare needs that often require transitions across multiple organizations. This study explores the experiences of diverse healthcare professionals from organizations involved in transitions between nursing homes and emergency departments and identifies conditions necessary to improve this transitional process.

**Methods:**

Eighteen qualitative interviews with healthcare professionals were conducted, and data were analyzed using thematic analysis based on the work of Braun & Clarke. This paper adheres to the Standards for Reporting Qualitative Research (SRQR) standards.

**Results:**

Three themes were identified: (1) *Inclusive and supportive engagement*, emphasizing the importance of involving care recipients while providing support; (2) *Operational readiness*, indicating the structures and supports needed to ensure that staff can effectively respond to unexpected events; and (3) *Cross-organizational collaboration*, pinpointing that streamlined communication strategies across healthcare organizations facilitate a shared decision-making process among healthcare professionals and ensure that essential patient information is readily accessible at each transitional step.

**Conclusions:**

A comprehensive approach addressing these individual, operational, and systemic factors can enhance transitional care between nursing homes and emergency departments.

## Background

Nursing home residents often experience acute illnesses, requiring frequent use of emergency medical services due to their heightened vulnerability and complex health conditions [1, 2]. These acute episodes can rapidly escalate during emergency department visits, risking both mental and physical deterioration [3]. Patient safety is at risk during transitions between nursing homes and emergency departments (NH-ED) due to factors such as communication breakdown, medication errors, cognitive impairment, resource scarcity, and lack of or inadequate treatment plans [4]. NH-ED transitions are time- and resource-intensive [5, 6], placing substantial strain on staff members, residents, and close family [7]. Furthermore, most European countries and the USA lack identified strategies regarding transitional care targeted toward NH residents [8].

Approximately 30% of NH residents in Norway utilize emergency healthcare services yearly, accounting for 20% of ED admissions [9]. Administered by municipalities, out-of-hours primary care (OHPC) manages urgent but non-critical cases outside regular hours, including NH-ED transitions when NH physicians are unavailable. Medical responsibility usually lies with a general practitioner (GP). However, when the NH physician is unavailable, particularly after hours, the OHPC physician becomes the primary decision-maker. In such cases, the OHPC staff will assess and determine the next steps, which can include an ED transfer. Registered nurses play a key role in identifying acute care needs and are often the first to contact a physician. Their assessments frequently include transfer decisions. Communication between organizations during NH-ED transitions primarily occurs digitally across different platforms, but telephone contact is also used when needed. Handoffs require a discharge summary, nursing report, and an updated medication list. Norwegian policy papers call for better collaboration between organizations caring for older adults by establishing protocols [10, p. 79, 11, p. 153, 12, p. 53, 13]. However, specific guidelines to achieve these recommendations are lacking, leading to varied protocols both within and across regions [9]. The 2012 Coordination Reform aimed to improve collaboration between primary (NH and OHPC) and specialist care (ambulance (AMB) and ED) by promoting more integrated and coordinated patient pathways [14]. However, concerns remain as both ED transfers and mortality rates after discharge have increased since the reform, reflecting more uncertain care needs among NH residents [15].

The existing body of knowledge reveals a challenge in care provision, as healthcare professionals must provide advanced care in a complex, multiorganizational healthcare setting. This situation raises concerns about whether healthcare delivery sufficiently addresses the needs of NH residents in transition and whether additional improvements are needed to ensure their safety and well-being. There is a need to identify ways to optimize these transitions, manage resources more effectively, and improve patient care outcomes. However, most initiatives to improve NH-ED transitions are location-specific [16], highlighting the need for a multidimensional and coordinated approach to ensure continuity of care [17]. A significant number of studies focus on specific phases or stakeholders, often neglecting the broader system and how different elements interact [18]. Few studies explore the perspectives of healthcare professionals, specifically what they believe makes an improved transition, which hinders the development of strategies in NH-ED transitions [19]. This paper aims to explore the unique experiences and conditions identified by healthcare professionals as essential for improving the NH-ED transitions. Ultimately, we ask the following question: *What are the necessary conditions to improve transitions between nursing homes and emergency departments according to healthcare professionals?* To address this, we apply transition theory as a theoretical framework and use thematic analysis as developed by Braun and Clarke to identify patterns in the data.

### Theoretical framing

**Fig. 1:**
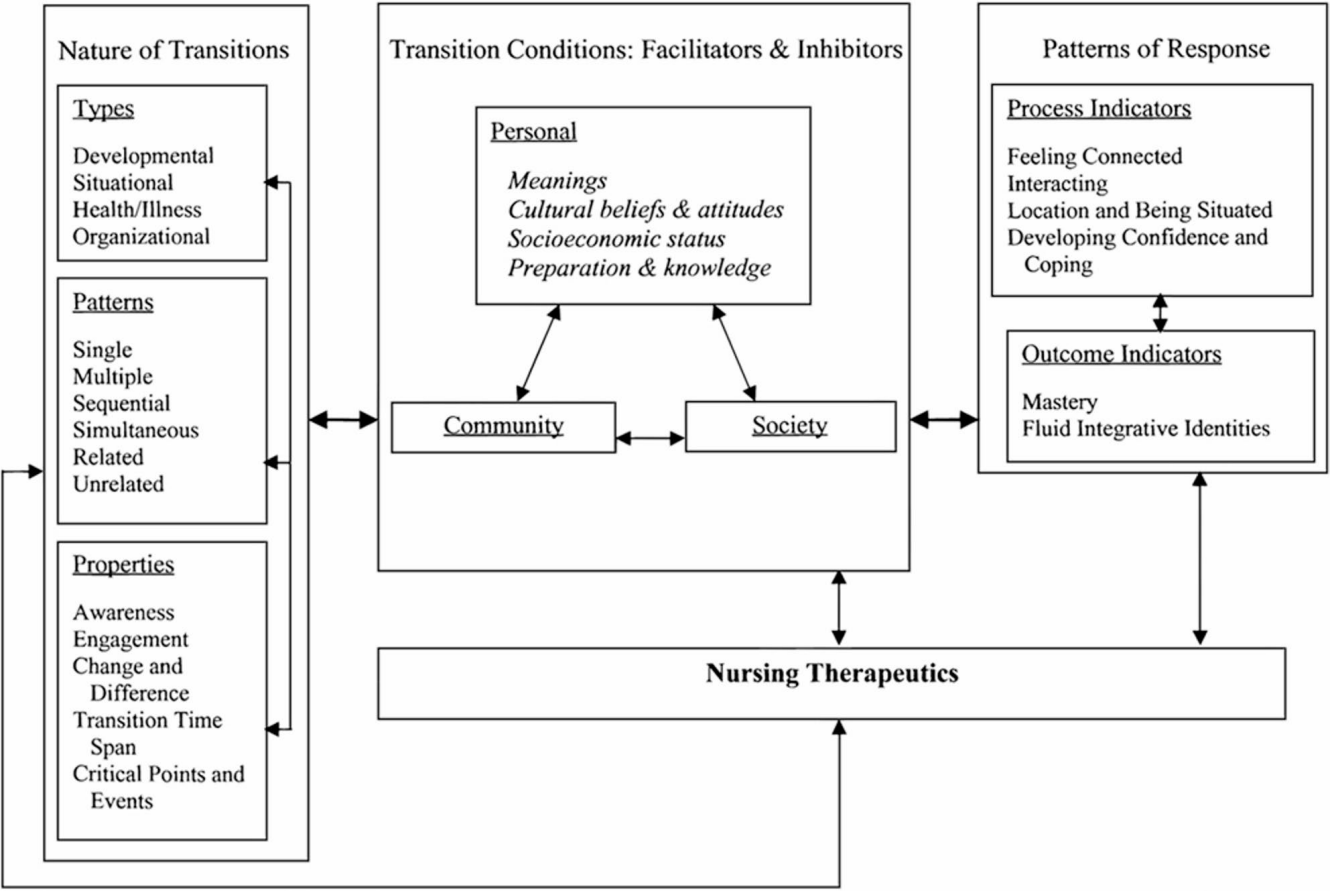
Transition theory

This paper utilizes transition theory framework articulated by Meleis et al. [27] to better understand the complex and multifaceted nature of transitions (Fig. 1). A *transition* is a dynamic and often nonlinear process that embodies personal, social, and cultural factors, whereas a *transfer* is the physical movement of an individual from one care setting to another [20]. Applying the concept of transition is relevant to our study, as we include the multifactorial processes in NH-ED transitions rather than looking at individual aspects in isolation. Healthy transitions occur when individuals successfully adapt to life changes while receiving support and guidance [21]. We approach the notion of healthy transitions as a sensitizing concept that provides a general sense of reference and guidance rather than clear definitions with precise criteria [22]. The framework was included in the development of the interview guide and to inform the discussion, which will be explained in more detail in the method and discussion sections.

 [23] Used with permission from Wolters Kluwer Health, Inc., License No. 6092930220859.

## Method

This study complies with the Standards for Reporting Qualitative Research (SRQR) [24] checklist to ensure transparency and trustworthiness. Qualitative interviews were conducted individually with healthcare professionals using a semi-structured interview guide with open-ended questions to explore their individual and unique experiences [24]. Transition theory and existing research informed the interview guide, which facilitated in-depth responses and follow-up questions. For example, as transition theory addressed the importance of information, we asked the participants to reflect on their experiences with providing and receiving information.

Pilot interviews were conducted, and the interview guide was refined afterward to ensure clarity, content validity, and feasibility. Some questions were too general, and follow-up questions were added. Online interviews were conducted from January to June in 2023 to streamline and simplify the recruitment and interview process [25]. All participants were invited for follow-up interviews from June to September in 2024, with eight of them agreeing to participate. The remaining participants were either unavailable or did not respond to the invitation. Multistage interviews were conducted to obtain a deeper understanding by reflecting on existing data [26]. During the reinterviews, eight of the 18 participants shared their thoughts and experiences since the initial interview, followed by a brief synopsis of their first interview to ensure that important issues were addressed. Data from both initial and follow-up interviews are included in the analysis. The first round of interviews was conducted until the absence of new evidence or data was sufficient to answer the research questions, following the recommendations of Green & Thorogood [27]. The dataset from the initial interviews was also used in a previous paper where the interview method and guide were first described in detail [7].

We compiled a list of all municipalities within Western Norway, and randomly selected managers from different organizations using numbered entries in the list. Managers from rural and urban regions of Western Norway then recruited 18 staff members from primary (NH and AMB) and specialist (OHPC and ED) healthcare (See Table 1). This stratified random sampling was conducted to identify groups of individuals aligned with the research question [28], which facilitated a more targeted recruitment process, ensuring the selection of participants with relevant knowledge and experience. Inclusion criteria were healthcare professionals with two years of work experience from NHs, AM services, OHPC offices, and EDs.

**Table 1:** Sample information

Location	Profession	Work experience	Specialty
NH	5 RN* and 1 GP*	6–39 yrs	Infection prevention and control (1 RN) Geriatric (1 RN) Gastroenterology (1 GP)
OHPC	3 RN	10–13 yrs	Acute medical care (1 RN)
AMB	1 AMB* and 2 RN + AMB	8–20 yrs	
ED	5 RN and 1 RN + AMB	4–30 yrs	Intensive care (1 RN) Acute medical care (2 RN) Anesthesia (1 RN)

* RN = Registered nurse, GP = General practitioner, AMB = Ambulance worker (Vocational certificate)

Using reflective thematic analysis developed by Braun & Clarke [29], six steps were followed to systematically identify, analyze, organize, and report patterns within the data. Through familiarization, the first author transcribed and revisited the data several times. Also, data were reduced and categorized to generate initial codes in Nvivo software from reoccurring topics. After reviewing the data, the first author searched for and documented themes. All authors collaboratively reviewed, assessed, redefined, and, where necessary, merged these themes, with the first author conducting a repeat data review to refine the final themes. Disagreements and differences of opinions were welcomed and reflected in the author team. Subsequently, final themes were defined and named. The research team then reviewed, discussed, and reached a consensus on these themes. The thematic analysis concluded with the production of the report, as demonstrated in the results section. According to Braun & Clarke [30], a potential weakness of thematic analysis is misunderstanding the essence of the themes. To enhance the rigor and reliability of the findings and avoid misinterpreting the themes, the analysis involved repeated engagement with the data, combined with a literature review and author team discussions. Furthermore, the main author wrote fieldnotes and kept an analysis journal to ensure reflexivity, transparency, and traceability throughout the analytic process. The author team has professional backgrounds in healthcare and qualitative research, and brought different perspectives to the analysis of data. This process also included reflecting on our own positionality, particularly how experiences and proximity to the field might have shaped the data interpretation.

### Research ethics

Our study was conducted in accordance with the guidelines of the Declaration of Helsinki [31], and ethical approval for the research was obtained from the Norwegian Agency for Shared Services in Education and Research (Ref. 2689669). Informed consent for participation was obtained verbally upon recording after repeating previously shared written information addressing the interviewees’ rights as participants. They were also informed that consent could be withdrawn without further explanation or consequences. The audio recordings and verbatim transcripts were stored in a secure research database to ensure the confidentiality of the participants.

## Results

The participants of this study shared experiences and outlined preferred practices they believed would foster improved NH-ED transitions. The data only showed minor variations in opinions among the healthcare organizations. Analysis of data identified the following three themes that point toward potential improvement: (1) inclusive and supportive engagement, (2) operational readiness, and (3) cross-organizational collaboration (See Table 2).

**Table 2:** Themes, subthemes, and selected quotes

Theme	Subtheme	Selected quotes
Inclusive and supportive engagement	Involvement of NH residents and close family	“*Of course*,* they should have an opinion about what happens*,* right?*” (OHPC staff).
	Support to NH residents and close family	“*We basically have to sit down and hold their hand. We spend much time doing that*,* to try and provide reassurance as best we can*” (ED staff).
Operational readiness	Preparedness	“*If there is a good plan there*,* it usually goes very well*,* because the NH have clear things in the patient journal that they can convey to the physician.”* (AMB staff)
	Sufficient staffing and equipment	“*You actually have to let go of what you are doing. Unless there is an acute need for you somewhere else*,* you actually have to come up there. We`re talking about putting out fires rather rapidly.*” (OHPC staff).
	Proper staff competency and training	“*It’s about updating knowledge and being aware of the responsibility that competency matures and evolves over time.*” (NH staff)
Cross-organizational collaboration	Adequate communication across healthcare organizations	“*We know many of the people who work there*,* and they know us*,* which kind of makes the communication easier*,* I think.*” (NH staff).
	Accessible and adapted patient information	*“I think it`s important for us to write really good patient journals also because they have to go through many different people in the system”* (AMB staff).
	Shared assessment and decision-making	“*We are really good at that. As soon as there are more people at work*,* we grab them*,* and then we do the assessment together.*” (NH staff)

### Inclusive and supportive engagement

#### Involvement of NH residents and close family

The participants reflected on the importance of inclusive and supportive engagement of NH residents and close family. The healthcare professionals were unanimous about actively including the thoughts and opinions of NH residents and close family. However, some participants shared that important details were sometimes missing or overlooked during transitions, which could hinder effective person-centered care. According to many participants, accommodating the needs and requirements of NH residents and their close families during NH-ED transitions was essential, yet sometimes missing. “*But the most important part of the transitional process is to take care of them*,* right? Provide information. Tell them what is happening. Reassure them. Some of them are scared*,* right?*” (NH staff). Several participants pinpointed the importance of taking a holistic approach to the individuals. This meant looking beyond immediate medical care, especially in an environment where urgent medical treatment is the focal point: “*You`re supposed to see the entire human as a whole. But in the ED*,* OHPC*,* you`re very focused on the medical stuff first.*” (OHPC staff). According to the participants, feedback was also considered a helpful part of transitional care. It was requested simply by asking the NH resident or close family: “*When they are done at the hospital*,* we always have a dialogue. How are you doing? How are you feeling now?*” (NH staff).

Many participants stressed the benefits of including close family members: “*They are so helpful because they calm the patient*,* and we receive good information that is useful to make decisions.*” (ED staff). Close family inclusion was particularly imperative when the resident had cognitive impairment or dementia. If NH residents were unable to participate actively or did not have a support system to advocate for them, many participants felt it was crucial to consider still the resident`s needs and preferences: “*It`s at least important that we`re sort of the patient`s attorney.*” (OHPC staff). Including individuals was difficult in urgent situations, but the participants shared examples of how they tried to improve the transition by clarifying events as they occurred: “*Yes*,* we describe what we`re going to do*,* where we`re going*,* and why we`re going there.*” (AMB staff). The participants shared several ways to include close family, such as making sure their basic needs are well taken care of: “*We can offer them the chance to sit next to the patient back with us if they want*,* if there are some concerns*,* or they believe it`s best for the patient.*” (AMB staff). The support from close family was not only beneficial to the NH resident but also to staff members, as they could attend to pending tasks: “*I consider close family to be a resource to the patients when they are here*,* and I have positive experiences with them being here.*” (ED staff). The participants also experienced that close family helped provide tailored care, although gaps in planning and communication often made it challenging to deliver fully personalized support.

#### Support to NH residents and close family

Providing support to NH residents was considered a key element of inclusive and supportive engagement. Several interview responses demonstrated how staff members tried to comfort the NH resident: “*We basically must sit down and hold their hand. We spend much time doing that*,* to try and provide reassurance the best we can*” (ED staff). Facilitating positive interactions was also considered to improve NH-ED transitions: “*And it`s essential*,* I think*,* to establish a relationship in a short time. I mean*,* we come into the room and make advanced treatments very quickly.”* (AMB staff).

### Operational readiness

#### Preparedness

The participants emphasized the importance of being fully prepared to effectively execute specific tasks and respond to operational demands, ensuring that resources, personnel, and processes were in place and ready for action. Preparedness often started with a conversation at the NH between staff members, NH residents, and close family: “*I think it`s important for the physician to have that conversation with the patient and close family when they move into the nursing home.*” (OHPC staff). These meetings should result in a plan that addresses their preferences in an emergency. The participants stated that a written plan was needed to identify the level and intensity of treatment necessary for the NH resident and close family. However, participants experienced that such plans were sometimes missing, leading to challenges in coordinating care during acute situations. During these meetings, close family played a pivotal role in the discussion, as they could supply information while also speaking on the residents` behalf. An emergency care plan was considered essential for promptly and effectively managing the acute illness, as it facilitated more effective communication of important information: “*If there is a good plan there*,* it usually goes very well*,* because the NH have clear things in the patient journal that they can convey to the physician*” (AMB staff).

#### Sufficient staffing and equipment

Sufficient staffing in times of urgency was pointed out in the interviews as significant to the success of NH-ED transitions, which were identified as resource-intensive: “*It is good to have more people at work because we can help each other*.” (NH staff). Several participants also wished for an improved workforce distribution in urgent situations to ensure that everyone was allocated where it was needed: “*You actually have to let go of what you are doing. Unless there is an acute need for you somewhere else*,* you actually have to come up there. We`re talking about putting out fires rather rapidly.*” (OHPC staff). However, some local variations could impact the experience of NH-ED transitions, such as geographical location, funding, equipment, staff competency, staff training, physician availability, passionate individuals at work, and other local conditions. “*A neighboring municipality is bigger*,* and [OHPC] is only necessary at night*,* whereas the other surrounding municipalities have OHPC available around the clock*” (OHPC staff). This could be positive or negative, depending on what resources were available. For example, one NH was located right above the AMB service building, and NH staff could easily consult the AMB staff for decision support. Other organizations were geographically remote or difficult to access, where one NH staff mentioned they had to operate very independently in urgent situations.

#### Proper staff competency and training

Another recurring topic was the need for competency and proper training to improve NH-ED transitions: “*All of this requires that there are people with the necessary expertise and that you have enough staff to make the right assessments*,* right?*” (OHPC staff). The healthcare professionals generally wanted more training on the acutely ill geriatric patient. A satisfactory level of competency and training was said to increase the staff members` confidence when dealing with acutely ill NH residents. “*Being confident and trusting oneself may seem terrifying to many. It’s about updating knowledge and being aware of the responsibility that competency matures and evolves over time.*” (NH staff). Participants reported that proper use of assessment tools improved NH-ED transitions: “*Instead of explaining how the patient is with many words and explaining blood pressure and pulse and all that*,* NEWS [an assessment tool to monitor clinical condition] can say something about how the patient is doing using a few words.*” (NH staff). However, all staff members needed proper knowledge and training for these tools to be useful: “*I am trying to implement it so that everyone in the municipality has the same too. And I think that is important for the cooperation to go swiftly.*” (NH staff).

### Cross-organizational collaboration

#### Adequate communication across healthcare organizations

All participants highlighted the importance of cooperation, which included adequate and efficient communication and information-sharing across healthcare organizations. A collaborative work environment facilitated a shared discourse of how to address challenges, as shared by a number of participants: “*That we have good cooperation and a shared understanding of how to solve the assignment*” (AMB staff). NH residents and close family should be included in the communication process to ensure that patient information is available. Communication methods should include the facilitation of meetings between staff members from the healthcare organizations mentioned in this paper. Such meetings were not always standard practice, and staff members lacked information about the residents` preferred care options. Healthcare professionals also sought more straightforward and effective ways to communicate with staff members from other healthcare organizations, as current ways of communicating were often complicated, time-consuming, and inefficient.

#### Accessible and adapted patient information

An essential aspect of cooperating in NH-ED transitions was access to relevant patient information, which enabled receiving staff to prepare better and ensure a smoother transition of care: “*What has been going on at the hospital? So that we can prepare a little. That way*,* we can stay ahead of what we can provide in terms of care and treatment afterward.*” (NH staff). Other helpful information could include possible updates on future plans: “*What is the plan moving forward*,* or where is he going next? Does he come back*,* or where does he go?*” (NH staff). In many cases, close family needed to relay information on behalf of NH residents who were unable to communicate, and access to the patient`s preferences was imperative: “*How should he lie on a gurney*,* how should he lie in bed. So yeah*,* maybe you need someone to assist that knows the patient.*” (NH staff).

#### Shared assessment and decision-making

The participants highlighted the importance of a thorough, shared assessment and decision-making across multiple healthcare organizations:

*We go out and do our measurements*,* and our assessments… our examinations and relay those to OHPC. And then we agree where the patient goes…whether we should stop by OHPC or go directly to the hospital or just stay there.* (AMB staff)

Most healthcare professionals wanted a shared response to acute illnesses where the organizations work together to share responsibilities during NH-ED transitions. A shared prehospital assessment and decision-making could facilitate a supportive environment for the staff members involved, where they did not feel isolated during the transitions. The ability to consult with other healthcare professionals could also boost staff confidence when uncertainties about the best approach arose: “*If you sort of feel that you have done something right or wrong*,* to have open discussions and reflection. At least the ethical ones. I think we should get better at that.*” (OHPC staff).

## Discussion

This paper outlines critical conditions needed to improve NH-ED transitions based on the experiences of healthcare professionals. Our findings highlight several interrelated themes that point towards a complex interplay between individual, relational, and systemic factors. While participants articulated a clear desire to improve the transitions, many of the practices described were informal and lacked systematic integration into routines. Meleis` transition theory serves as the primary theoretical lens to interpret the data, but we also draw selectively on the Consolidated Framework for Implementation Research (CFIR) to help interpret findings. Specifically, the themes identified from the analysis manifest at three levels: “characteristics of individuals” (personal attributes), “inner settings” (organizational factors), and “outer settings” (systemic influences) [32, 33]. These factors are less directly addressed by transition theory. Consequently, CFIR provides a valuable structure for interpreting our findings by illuminating how broader systemic and organizational issues affect individuals.

### Inclusive and supportive engagement

First, our findings emphasize the importance of inclusive and supportive engagement, specifically the involvement of NH residents and close family. This approach aligns with the CFIR domain “*characteristics of individuals*,” which emphasizes that innovations are shaped by the knowledge, beliefs, and preferences of individuals [32, 33]. Our findings suggest that efforts to improve NH-ED transitions should actively consider the perspectives of individuals. To enhance emergency services for older adults, it is essential to engage care recipients in decision-making [34], provide clear and understandable information, offer tailored care [44], and involve patients in the decision-making process [35]. Transition theory underscores the importance of active participation in transitions, as disruptions can threaten the autonomy of an individual and undermine the individual’s sense of autonomy and control [36]. However, older adults often hesitate to seek information, which should prompt healthcare professionals to encourage an active dialogue personalized to the capacity of the older adult [37]. Moreover, older adults and close family often have a heightened need for warm and nurturing encounters with staff members during NH-ED transitions [7]. Our findings and existing research indicate that involving NH residents and their families is not just a clinical necessity but also an emotional matter, given the psychological impact of urgent events on NH residents. Healthcare professionals, older adults, and close family in transition need tailored information and support. However, this study adds that healthcare professionals struggle to adjust the complexity and format of information to meet individual needs, especially when cognitive impairment is present. We suggest that healthcare professionals actively encourage NH residents and close family to communicate openly by welcoming questions, addressing concerns, and cultivating discussions to promote mutual understanding and build trusting relationships in transitions.

The participants also emphasized the importance of establishing positive relationships in a short period of time. As older adults feel anxiety and vulnerability in urgent healthcare situations, they rely on healthcare professionals for support [35]. However, emergency departments often prioritize medical treatment [38]. Moreover, healthcare professionals often experience shortcomings in accommodating personal needs, leading to feelings of neglect among NH residents and close family [39]. Gjerstad et al. also point out that building trust with older adults and close family in an acute setting needs to be systemic (financing, frameworks, and infrastructure) and individual [40]. The 4M framework is a guiding model which is developed for age-friendly healthcare, where a key priority is empathetic communication by asking older patients what matters to them while using compassionate language. Such age-friendly practices are meant to encourage trusting relationships between care providers and recipients [41]. The components from the 4M framework align with our findings, and demonstrate ways to support individual needs through a systemic imperative. Transition theory states that positive relationships foster opportunities for personal development and self-actualization. As transitions can disrupt relationships, fostering a sense of connectedness through engagement with others becomes pivotal [36]. This pinpoints the need to prioritize holistic care that addresses older adults’ physical and emotional needs. Our findings also indicate that staff members seek support and positive relationships from other staff members as they are an integrated part of the NH-ED transitions. However, the time-restricted and work-intensive environment in urgent care can challenge healthcare professionals in doing their jobs. As a result, they rely on the support of their peers and collaborative networks to navigate the high demands of their roles and ensure proper patient care, making positive relationships increasingly important to healthcare professionals.

### Operational readiness

Second, our findings indicate the need for operational readiness, which includes being prepared for unexpected events in order to enhance NH-ED transitions. According to the CFIR framework, successful interventions in the “*inner setting*” domain are linked to a positive work culture where staff members can respond quickly to changes [32, 33]. This aligns with our findings, suggesting that transitional care for older adults requires adaptability to address the diverse and evolving challenges of patient care. In line with this assertion, healthcare professionals need departmental capacity to operate flexibly. Therefore, care pathways should make room for adjustments to respond quickly to evolving patient conditions when necessary. Our findings are similar to those of Lemoyne et al., who identify that acute care for older adults should include clear directives on issues such as non-hospitalization requests and do-not-resuscitate orders [1]. Prehospital protocols should reduce risk among older adults, encourage hospitalizations for thorough assessments, and give accurate diagnoses [42]. The level and type of preparation required for healthcare transitions depend on the services available and the specific needs of individuals [43].

However, the lack of advanced care directives among older adults often leads to unnecessary hospitalizations and care initiatives that fail to align with the patient’s wishes [44]. One way to address this issue is through the systematic use of Advanced Care Planning (ACP), which enables older adults to articulate their preferences for future healthcare in the event of a health crisis. ACP supports shared decision-making and enhances person-centered care [45] and can improve the inter-organizational communication by ensuring that individual preferences are communicated and shared across different settings [46]. ACP is also beneficial for the physical and psychosocial health of older adults in need of emergency hospitalizations [47]. Thus, ACP holds the potential to clarify emergency plans and strengthen coordination across levels during NH-ED transitions. However, recent studies have shown a low level of implementation of ACP in clinical practice [45, 48, 49]. Our findings add that the absence of such structured planning contributes to uncertainty and reactive decision-making in the process. This resonates with transition theory, which identifies preparation, engagement, and available support as essential conditions for promoting healthy transitions [23]. In this context, ACP can be viewed as a specific measure for critical points in NH-ED transitions.

It was also crucial for the participants to collaborate on patient assessments and decision-making. However, the complex needs of older adults were mirrored in the unique and unpredictable tasks of healthcare professionals, making it difficult to collaborate during the process. Given that NH-ED transitions are more work-intensive than regular tasks [50], exploring ways to allocate available resources to manage them effectively is also essential. O`Donnell et al. [51] state that decision support and adequate resources, including funding and staffing, are necessary to implement successful transitional care. Thus, we recognize the need for initiatives to ensure proper use of resources. Other research suggests that healthcare professionals should be able to provide both acute care and geriatric assessment to patients with acute illness [52]. These findings can be further understood through the lens of transition theory, which refers to role insufficiency, where care providers are unable to effectively respond to the expectancies of their role or context [23]. In the context of this paper, this concept helps explain how care providers may feel unequipped to provide transitional care without sufficient timing, training, or systemic infrastructure. Ultimately, care providers need the necessary skills and additional training to recognize and manage acute illnesses in older adults.

### Cross-organizational collaboration

Third, our data analysis indicates the need for cross-organizational collaboration, which involves improved communication, sharing of information and decisions, and mutual support among healthcare organizations. This suggests that transitional care should be a shared responsibility rather than left to the individual. The CFIR framework domain “*outer settings*” includes broader system-level components, such as inter-organizational partnerships, policies, funding, and organizational structures. The framework pinpoints explicitly that external factors, to a large degree, impede or facilitate implementation [32, 33]. Our data supports the framework and suggests a need for strategies that actively promote engagement in shared responsibilities, discussions, and decision-making between healthcare professionals across organizations. Other researchers support this assertion by stating that the healthcare transitions of older adults should include close collaboration between primary and specialist care [51]. Moreover, urgent care provision to older adults should consist of decision support [53]. Furthermore, a collaborative approach benefits NH residents and close family through coordinated and comprehensive care [6]. This underscores the need for a team-based approach to NH-ED transitions, where interpersonal teamwork and cross-disciplinary collaboration are prioritized. However, this does not seem to reflect current practices where healthcare professionals often make decisions without access to helpful communication tools or the support of their peers. Cross-organizational collaboration in NH-ED transitions is significant because it includes multiple care settings. Also, Moore et al. identify several barriers to collaboration between municipal healthcare and hospital services, including rigid professional structures, limited time and resources, unclear role boundaries, and fragmented organizational structures. Such barriers hinder effective information-sharing, cause frustration among healthcare workers, and ultimately diminish their motivation to collaborate with healthcare professionals from other organizations [54]. This aligns with our findings, indicating that care providers often lack both systemic support and operational clarity. As a result, improvement efforts remain informal, under-resourced, and inconsistent. The need for a shared responsibility is also present in transition theory, which highlights the presence of support and systemic alignment across contexts [23]. Thus, achieving healthy NH-ED transitions requires more than informal collaboration, as it calls for structural support and shared responsibilities across organizations.

Our findings indicate that a significant aspect of collaboration is building connections with other healthcare professionals and ensuring that decision-making processes are inclusive rather than leaving individuals to navigate complex transitions in isolation. Hedqvist et al. [55] emphasize that cross-organizational collaboration requires sharing information and decisions, enhancing communication, and fostering mutual support among healthcare organizations. Transition theory points out that positive transitions include a sense of connectedness and belonging, which entails receiving support and being a part of a larger group [21]. However, our data suggests that these relational conditions are not consistently present, partly due to unclear role boundaries, siloed organizational structures, and the absence of structured forums for joint decision-making. Limited time, high workloads, and a lack of effective communication pathways may hinder integrated work practices. Future efforts to improve NH-ED transitions should address systemic barriers and foster reciprocal collaboration, enabling healthcare professionals to communicate and share information effectively while supporting each other. We recognize that the participants of this study rarely addressed how interprofessional relationships influence the experience of NH-ED transitions. Geese identifies several benefits of interprofessional care in transitions: streamlined care coordination, bridging care gaps, education and training, efficiency in care delivery, cost savings, and better patient outcomes [56]. Muller et al. [57] add that interprofessional collaborations can facilitate improved communication. However, interprofessional teamwork can be hard to maintain over time and should be managed systematically to ensure fidelity [58]. Our findings do not necessarily point out that interprofessional collaboration is insignificant in NH-ED transitions, and existing research shows the opposite. Instead, our study could demonstrate a lack of interprofessional culture among our participants. There is a desire and willingness among these professionals to cooperate, but our findings indicate that they lack the tools to implement a cross-organizational collaborative culture targeted toward NH residents with acute illness. This would suggest that any efforts to implement interprofessional collaboration should be systematically embedded through routines and standardized practices to ensure that healthcare professionals are aligned in their approaches. Examples from Azzelino et al. [59] suggest standardized efforts that include post-discharge procedures, case coordination models, and integrated collaboration between different healthcare professions. Similarly, Marini et al. [60] emphasize that structured follow-up and caregiver involvement can improve transitional care, which can be translated into NH-ED transitions.

### Close family involvement

A recurring theme was the critical role of involving close family members, as they provided emotional support to the NH resident while also improving communication by facilitating the exchange of information between older adults and staff members. Fry et al. [61] refer to family members as “safety nets” as they provide advocacy, support, and patient information. This underscores the notion that close family play a pivotal role in improving the continuity of care while advocating for their loved ones and supporting the healthcare professionals in information-sharing. Transition theory also emphasizes the importance of close family, as they are a pivotal part of the relational environment that either facilitates or hinders healthy transitions [23]. As such, ensuring that close family receive support and are integrated into the care practice is essential to a person-centered care in transitions.

Furthermore, we see the need to streamline communication between healthcare staff and residents, as well as between healthcare organizations. However, one paper specifically points out that the opinions of close family might differ from those of healthcare professionals and NH residents. Moreover, being involved can be stressful to close family members [62]. Thus, involving close family in NH-ED transitions could potentially leave family members with responsibilities beyond their capabilities or wishes and may point toward insufficiencies in transitional care. Therefore, it is essential to approach this matter with sensitivity and support, considering the needs and preferences of close family to prevent burnout. This is mirrored by Malhotra et al. [63], stating that ACP directives can alleviate caregiver burden by clarifying patient preferences, improving communication, thus reducing emotional stress and enhancing preparedness. Collectively, these insights demonstrate the need for structural support to safeguard not only NH residents, but also their close family.

### Putting insight into action

It is noteworthy that our data does not present specific interventions. Høyvik et al. identify that there are few routines targeted specifically toward older adults in NH-ED transitions, with most strategies being similar to those for the general population [7]. Although the participants in this paper were asked direct questions such as “How do you think one should cooperate with other healthcare organizations?”, their responses placed greater emphasis on preferable conditions than specific strategies. A few examples were given of local initiatives set in motion to improve current practices during these transitions, such as teaching procedures to their co-workers through spontaneous workshops. These efforts were not necessarily implemented and governed by management as part of their routines, suggesting a lack of systematic integration into standard protocols. This may point to a broader issue where healthcare professionals face challenges in clearly expressing or putting into action plans to improve NH-ED transitions, and we have concerns that healthcare professionals lack the opportunity for shared reflections within and across healthcare organizations. One study points out that healthcare transitions often lack operational clarity [43], which resonates with our findings that healthcare professionals have a clear vision of their goals but struggle to define the pathway to achieve them. A possible reason could be that the challenges at hand relate to higher-level structures, such as staffing, training, communication tools, etc. Transition theory adds that healthy transitions include structure, preparation, and role clarity as key conditions for healthy transitions [23]. The absence of standards and protocols creates uncertainties, as care providers navigate transitions without sufficient guidance. As such, there needs to be a stronger focus on developing shared routines and structural support that translate insights from care providers into actionable and coordinated practices.

### Implications for practice

In summary, improving NH-ED transitions requires interventions that address both systemic and relational aspects of care. Based on these finding, future practices should facilitate standardized care pathways, formalizing cross-organizational communication pathways, and encourage staff training. Such procedures should encourage care provider support, so that they can fulfill their intended purpose.

### Limitations of the study

The sample of healthcare professionals, primarily nurses, may not fully represent all professions involved in NH-ED transitions. However, their insights were crucial as they play a key role in facilitating these transitions, which could explain why they made up the bulk of our sample. Also, the nurses represented various specializations (Table 1), indicating some diversity within this group.

The reinterview sample was smaller than anticipated, which could represent a potential limitation in the study, and a larger number of participants could have yielded a more comprehensive understanding. However, the reinterviews were valuable in providing more profound reflections on the topic.

A limitation of this study is the absence of data from NH residents and close family. Including their perspectives could provide valuable insights into NH-ED transitions and should be explored in future research. However, this study specifically focuses on the experiences and roles of care providers.

## Conclusion

Our study explores the perspectives and experiences of healthcare professionals. It highlights the need for a comprehensive approach addressing individual factors (*characteristics of individuals*), operational capabilities (*inner setting*), and systemic barriers (*outer setting*) in NH-ED transitions. The CFIR framework domains suggest that interventions should be tailored to challenges and opportunities by integrating multiple dimensions, which include improving care coordination, communication, and resource allocation. A holistic approach, informed by transition theory, incorporates stakeholder perspectives to enhance care for healthcare professionals, NH residents, and close family.

Improving NH-ED transitions requires fostering seamless communication, coordinating care, and adhering to safety and quality protocols while balancing standardized practices with contextual adaptations. This paper highlights systemic issues affecting patient care, emphasizing the need for structural changes and better support for healthcare professionals, enabling them to manage individual and local variations in these transitions.

## Data Availability

The datasets used and analyzed in this paper are available from the corresponding author on reasonable request.

## Abbreviations

AMB: Ambulance services
ED: Emergency department
NH: Nursing home
OHPC: Out-of-hours primary care
